# Introducing meta-services for biomedical information extraction

**DOI:** 10.1186/gb-2008-9-s2-s6

**Published:** 2008-09-01

**Authors:** Florian Leitner, Martin Krallinger, Carlos Rodriguez-Penagos, Jörg Hakenberg, Conrad Plake, Cheng-Ju Kuo, Chun-Nan Hsu, Richard Tzong-Han Tsai, Hsi-Chuan Hung, William W Lau, Calvin A Johnson, Rune Sætre, Kazuhiro Yoshida, Yan Hua Chen, Sun Kim, Soo-Yong Shin, Byoung-Tak Zhang, William A Baumgartner, Lawrence Hunter, Barry Haddow, Michael Matthews, Xinglong Wang, Patrick Ruch, Frédéric Ehrler, Arzucan Özgür, Güneş Erkan, Dragomir R Radev, Michael Krauthammer, ThaiBinh Luong, Robert Hoffmann, Chris Sander, Alfonso Valencia

**Affiliations:** 1 Structural Biology and Biocomputing Programme, Spanish National Cancer Research Centre (CNIO), C/Melchor F. Almagro 3, 28029 Madrid, Spain; 2 Bioinformatics group, Biotechnological Centre, Technical University Dresden, Tatzberg 47-51, 01307 Dresden, Germany; 3 Humboldt Universität zu Berlin, Unter den Linden 6, 10099 Berlin, Germany; 4 Institute of Bioinformatics, National Yang-Ming University, No. 155, Sec. 2, Linong St., Beitou District, Taipei City 112, Taiwan; 5 Institute of Information Science, Academia Sinica, No.128, Sec. 2, Academia Rd., Nangang District, Taipei City 115, Taiwan; 6 Department of Computer Science and Engineering, Yuan Ze University, 135 Yuan-Tung Rd., Chung-Li, Taoyuan, R.O.C., 32003, Taiwan; 7 Division of Computational Bioscience, Center for Information Technology, National Institutes of Health, Bethesda, Maryland 20892, USA; 8 Department of Computer Science, University of Tokyo, Hongo 7-3-1, Bunkyo-ku, 113-0033 Tokyo, Japan; 9 Department of Computer and Information Science, Norwegian University of Science and Technology, Sem Sælands vei 7-9, NO-7491 Trondheim, Norway; 10 Biointelligence Laboratory, School of Computer Science and Engineering, Seoul National University, Seoul 151-744, Korea; 11 Center for Computational Pharmacology, University of Colorado School of Medicine, P.O. Box 6511, Mail Stop 8303, Aurora, CO 80045-0511, USA; 12 School of Informatics, University of Edinburgh, 2 Buccleuch Place, Edinburgh, EH8 9LW, UK; 13 Text Mining Group, Medical Informatics Service, University and Hospitals of Geneva, 24 Micheli du Crest, 1201 Geneva, Switzerland; 14 Artificial Intelligence Group, University of Geneva, 7 route de Drize, 1227 Carouge, Switzerland; 15 Department of Electrical Engineering and Computer Science, University of Michigan, 2260 Hayward Street, Ann Arbor, MI 48109, USA; 16 Department of Pathology, Yale University School of Medicine, 300 Cedar Street, TAC 309, New Haven, CT 06520-8023, USA; 17 Program for Computational Biology and Bioinformatics, Yale University, Suite 501, 300 George Street, New Haven, CT 06520-8084, USA; 18 Computer Science and Artificial Intelligence Laboratory, Massachusetts Institute of Technology (MIT), The Stata Center Building 32, 32 Vassar Street, Cambridge, MA 02139, USA; 19 Computational Biology Center, Memorial Sloan Kettering Cancer Center, 1275 York Avenue, New York, NY 10065, USA

## Abstract

We introduce the first meta-service for information extraction in molecular biology, the BioCreative MetaServer (BCMS; ). This prototype platform is a joint effort of 13 research groups and provides automatically generated annotations for PubMed/Medline abstracts. Annotation types cover gene names, gene IDs, species, and protein-protein interactions. The annotations are distributed by the meta-server in both human and machine readable formats (HTML/XML). This service is intended to be used by biomedical researchers and database annotators, and in biomedical language processing. The platform allows direct comparison, unified access, and result aggregation of the annotations.

## Background

Information retrieval (IR), information extraction (IE), and text mining have become integral parts of computational biology over the past decade [1]. However, these services are dispersed, integrated in specific packages, and include proprietary software. Therefore, progress in the field requires offering better access to the tools, methods, and their results [2]. Other areas, such as sequence analysis, genome analysis, or protein structure prediction, have benefited greatly from enhanced access to services and tools for the community of biologists, bioinformaticians (through web servers and portals), and developers (by providing free, open source academic software) [3].

Web services, widely used throughout the internet to provide the functionality for distributed systems, are becoming a common part of bioinformatics tools; For example, one of the most used text mining applications, namely iHOP (Information Hyperlinked Over Proteins), provides such an infrastructure to access its data [4]. Meta-services, too, are a ubiquitous component of the world wide web, found as meta-search engines, in business-to-buisness and business-to-consumer transactions (for example, for flight booking systems), and are used in scientific research (for example, for protein structure prediction) [5]. Another example of a distributed meta-service is BioDAS (Distributed Annotation System), a platform to exchange biologic sequence annotations between independent resources [6].

This publication describes the development of the BioCreative MetaServer (BCMS) prototype. The Results section (below) provides an overview of the system design and introduces the basic components, followed by short descriptions of the IE systems currently available through the platform prototype. The Discussion section (below) reviews what problems are solved and what issues need further investigation. The Conclusions section (below) closes with current and future utilities of this platform for the biomedical community. Technical details on the platform and implementation aspects can be found in the Materials and methods section (below).

## Results

The fundamental aim of the BCMS platform is to provide users with annotations on biomedical texts from different systems. At the current prototype level, the dataset is restricted to a fixed number of approximately 22,800 PubMed/Medline abstracts. The available annotations consist of marking passages that are detected as gene or protein name mentions, annotating the articles with the gene/protein and taxonomic IDs (providing hyperlinks to the corresponding database entries), and a confidence score for whether the text contains protein-protein interaction information. Expanding on stand alone IE systems, this platform gathers the results of several systems developed by various research groups, unifies them, and allows the user to access abstracts and annotations in a combined view. It is conceivable that collating classification results will often enhance performance, simply because multiple equal classifications for a given annotation are more likely to be correct. The gathered data are accessible to the user both as human-readable hypertext and as machine processable XML in the form of XML-RPC requests.

### System design

The platform is to be regarded as a distributed system requesting, retrieving and unifying textual annotations, and delivering these data to the user at different levels of granularity. The BCMS can be divided into three main units.

• A static collection of text (a set of approximately 22,800 PubMed abstracts used in the BioCreative II challenge [7]).

• A set of active servers providing annotations for text (see Table 1 for participating servers) upon request; these annotation servers (AS) only interact with the meta-server and not directly with each user.

**Table 1:** Annotation servers

Team/Group	GM	GN	TX	PPI	Conf	State	Web Page
Hakenberg	+	+	+	+	True	Dynamic	
Kuo	+				False	Dynamic	
Tsai	+			+	True	Dynamic	
Lau		+			True	Dynamic	
Sætre	+	+		+	True	Static	
Kim				+	True	Dynamic	
Baumgartner	+	+		+	False	Dynamic	
Haddow	+	+	+	+	True	Static	
Ruch		+		+	True	Dynamic	
Özgür				+	True	Static	
Luong		+			True	Dynamic	
Hoffmann	^a^	+	+	+	True	Dynamic	
Totals	6	8	3	9	10	12 teams	

List of annotation servers used by the meta-server, plus a Boolean flag determining whether the classifiers use a confidence score (Conf) and the system state (State): dynamic = online system, already capable of delivering content for any PubMed abstract; static = offline system, server in development. The webpage columns provide a link to an online site for a team's annotation system. ^a^iHOP (Hoffmann) delivers GMs, but because of data compatibility issues this is a feature to be added in later versions of the meta-server. GM, gene/protein mention detections; GN, gene/protein normalizations; PPI, protein-protein interaction classification; TX, taxon classifications.

• A meta-server providing the combined data, namely both the annotations and the corresponding text. Therefore, users indirectly communicate with the annotation servers, using the meta-server as proxy.

**Figure 1:**
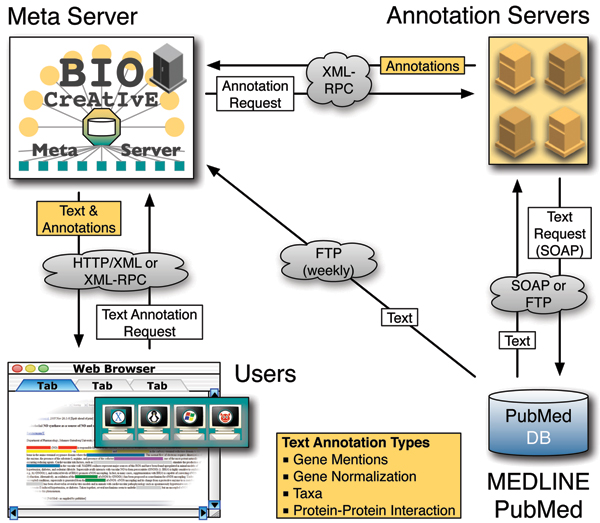
BioCreative MetaServer (BCMS) annotation view screenshot. This screenshot of the annotation view of the meta-server shows the main annotations for the given Medline abstracts (PMID 16458891). Central view: gene mentions (GMs) are marked in the text, ranging from gray (single annotation server [AS] detecting the particular mention) to yellow (all five ASs that have analyzed the text detect the highlighted text snippet as a GM), as a gradient that is shown below the text. At the bottom, the list of servers providing the annotations for this abstract can be found (only four of all thirteen visible). Left column: all raw annotation results can be viewed here. Gene mentions (GMs) results are expanded and sorted first by the number of servers predicting that mention and then by the median confidence for it. On the bottom left, a quick bar indicates protein-protein interaction (PPI) results. The bar is split in two, where the left and right bar lengths indicate the number of servers classifying this abstract as negative or positive in relation to mentioning PPIs. The color of the bars indicate the mean confidence of all classifications of one type: the negative (left) bar ranges from blue (low) to yellow (high confidence), and the positive (right) bar from yellow (low) to blue (high confidence). The bar also provides some interactivity: shortened names (indicated py an elipsis at the end) can be seen in their full form by mouse over, mousing over the gene mention highlights its position in the text, and individual gene normalization results can be clicked to see the exact database identifier, name, organism and a link to the DB record. Right column: by clicking on an italic mention in the central view, all possible mappings of GMs to GNs are shown: in bold the GM, and then the list of GNs (together with the species) and their official names (here for the text span "interferon-inducible p200 family"). This simple mapping is based on case-insensitive substring matching of GMs and the GN names and synonyms extracted from the DB records.

For all communication purposes, the system utilizes the XML-RPC protocol [8]. The meta-server sends requests to annotate a PubMed/Medline abstract to all known annotation servers. Once the ASs have finished processing the text, the annotation data are returned to the meta-server, which stores all annotations in its central repository. Whenever a user requests annotations for an abstract, the meta-server checks whether the annotation data already exist. If not, then it triggers a remote procedure call (RPC) with the PubMed ID to the ASs; otherwise, the server immediately returns the stored results to the client (Figure 1). There are two principal ways to access the meta-server: via web browser or by using the XML-RPC web service. In the former case, the results are asynchronously returned to the user via AJAX, whereas in the latter case - the web service - the response is sent once all results have been gathered. The system is intended to work at a maximum latency of about 10 seconds, after which the annotation servers are expected to have returned their annotation results to the meta-server. Obviously, these response times will increase under heavy load when many requests for non-annotated citations are made and will need constant monitoring. If the annotations for the PubMed ID have been generated already, the stored results are returned instantaneously for both (browser and RPC) scenarios.

The data can be provided by three different means, which also correlate with the three main components.

**Figure 2:**
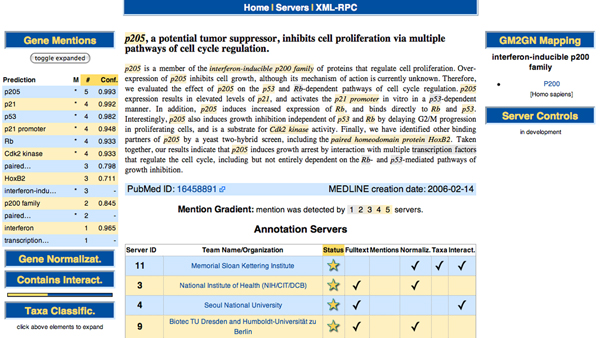
BioCreative MetaServer (BCMS) annotation view screenshot. This screenshot of the annotation view of the meta-server shows the main annotations for the given Medline abstracts (PMID 16458891). Central view: gene mentions (GMs) are marked in the text, ranging from gray (single annotation server [AS] detecting the particular mention) to yellow (all five ASs that have analyzed the text detect the highlighted text snippet as a GM), as a gradient that is shown below the text. At the bottom, the list of servers providing the annotations for this abstract can be found (only four of all thirteen visible). Left column: all raw annotation results can be viewed here. Gene mentions (GMs) results are expanded and sorted first by the number of servers predicting that mention and then by the median confidence for it. On the bottom left, a quick bar indicates protein-protein interaction (PPI) results. The bar is split in two, where the left and right bar lengths indicate the number of servers classifying this abstract as negative or positive in relation to mentioning PPIs. The color of the bars indicate the mean confidence of all classifications of one type: the negative (left) bar ranges from blue (low) to yellow (high confidence), and the positive (right) bar from yellow (low) to blue (high confidence). The bar also provides some interactivity: shortened names (indicated py an elipsis at the end) can be seen in their full form by mouse over, mousing over the gene mention highlights its position in the text, and individual gene normalization results can be clicked to see the exact database identifier, name, organism and a link to the DB record. Right column: by clicking on an italic mention in the central view, all possible mappings of GMs to GNs are shown: in bold the GM, and then the list of GNs (together with the species) and their official names (here for the text span "interferon-inducible p200 family"). This simple mapping is based on case-insensitive substring matching of GMs and the GN names and synonyms extracted from the DB records.

• Via web browser [9]. The main intention of this access method is to allow end-users (biomedical researchers) to search for a specific piece of information, e.g., to identify or confirm interaction partners for a given gene or protein. This view correlates with the meta-server unit (the third BCMS unit described above) and offers the user a graphical interface to explore the text and annotations (Figure 2).

• The second option is to use the XML-RPC protocol. This method is intended to provide developers with a means to integrate the platform data with their own applications, for example to use in combination with other annotation pipelines. Therefore, this is the direct interface to the ASs (the second BCMS unit described above), because the meta-server only acts as a proxy in this scenario. The API of the XML-RPC service can be found online [10].

• The third option is to contact the authors for a database snapshot of the current state of the meta-server data. This option is of interest for IE and text mining applications that make heavy use of the data, where online RPC would not be an option. This roughly correlates with the static content of the platform (the first BCMS unit described above). Because this is a rather crude access method, it might be improved (for example, a daily updated FTP download service) once the prototype stage is fully completed and enough interest is signaled.

### Annotation systems

Annotating biomedical abstracts can be done at various levels of granularity. Currently, the service provides four types of annotations.

• Gene/protein mention (GM): locate positions in the text that are detected as gene or protein names.

• Gene/protein normalization (GN): detect which genes or proteins are mentioned, assigning sequence database identifiers to the text.

• Taxon classification: identification of the organisms to which the text pertains, together with a confidence score, providing an ID for the National Center for Biotechnology Information (NCBI) taxonomic database.

• Protein-protein interaction (PPI): classifies whether the text contains PPI information and assigns a confidence score to the classification.

GM and GN may also provide confidence scores, depending on the annotation system. All confidence scores are normalized to the ]0, 1] range to render them directly comparable. For any given identical annotation between two or more annotation servers, the mean (to compensate for outliers) of the confidence scores is calculated. If an AS returns no confidence scores, then the result is not accounted for in the calculation of the mean. Note that the annotation systems employed here result from a recent challenge evaluation of the state of the art for such text-mining tasks [11]. According to the evaluation, gene mentions can be recognized with an F measure of more than 87% [12]. The gene name normalization has been shown to yield a top performance of more than 81% [13]. Classifications of whether a text discusses one or more protein-protein interactions can reach F measures above 78% [14]. Currently, there are 12 teams providing annotations for the meta-server. Here, a short overview of each system explains how results are generated (see Table 1 for an overview of the classifiers that each annotation server provides). The section titles (below) consist of the team locations and author initials in parenthesis, followed by the team name identifier used in Table 1 in square brackets.

#### Biotec TU Dresden and Humboldt-Universität zu Berlin (JH, CP) [Hakenberg]

The annotations we currently provide are gene mention normalization (32,795 human genes from EntrezGene), protein mention tagging (about 200,000 proteins from UniProt/SwissProt), NCBI taxonomy IDs for species mentioned in texts, and classifications of whether the text discussed one or more protein-protein interactions. Gene mention normalization was evaluated on the BioCreative II GM data and yields a precision of 79% at 83% recall [12]. Entity mention normalization is based on large lexicon of known names and synonyms, which are kept in main memory at all times for efficiency. Once a potential named entity has been found, we further identify it using context profiles in case multiple entities share the same name [15]; these profiles contain knowledge about each candidate entity, such as GO terms, chromosomal locations, or tissue specificity. We rank genes according to pieces of profiles also recognized in the current text. Annotations of proteins, species, and protein-protein interactions are based on the Ali Baba system [16]. Protein names are not normalized to a single UniProt ID, but potentially multiple IDs are returned for polysemous names. Recognition of species is currently based on about 200,000 names from the NCBI taxonomy. Ali Baba matches consensus patterns identified by multiple sentence alignment to recognize relationships between entities in texts. We decided to split the annotation services into two different servers: one for gene mention normalization and one for the other tasks. Please refer to the BCMS and the website mentioned in Table 1 for more detailed information on how to contact the services.

#### Institute of Information Science Academia Sinica (CK and CH) [Kuo]

Kuo and coworkers' system [17] based on conditional random fields (CRFs) for gene mention tagging, is among the best performing systems in this challenge evaluation. The key features of the system include a rich feature set, unification of bidirectional parsing models, a dictionary-based filtering, postprocessing, and its high performance. We carefully selected several feature types for CRF tagging, including character *n*-grams (window size 2 to 4) and morphological as well as orthographic features. In addition, we picked up several domain specific features (for example, biochemical terms such as cDNA, mRNA, tyrosine, and so on). On the other hand, some more commonly used features, such as stop words, prefix, and suffix, were not labeled. We utilized -2 to 2 as the offsets to generate contextual predicates. Then, we trained both forward and backward parsing models and combined them to obtain the final tagging results. This release is different from the version that we used to produce runs for BioCreative II. We still used MALLET [18] to perform CRF training and testing and Genia Tagger [19] for POS tagging, but we rewrote the feature extractors in Java and optimized the implementation to enhance greatly the efficiency. We also tuned the feature set to remove redundancies and other minor issues in the original feature set. As a result, this release can achieve a slightly higher F score than the original version with better efficiency.

#### Institute of Information Science (RT and HH) [Tsai]

Our annotation system [20] supports GM recognition and PPI text classification. For named entity recognition, we employ CRF as the underlying machine learning (ML) model; a set of features selected by a sequential forward search algorithm; numerical normalization; and pattern-based postprocessing [21] to help ML-based GM to deal with extremely difficult cases that need longer context windows. For PPI, we use a support vector machine (SVM) with a novel feature representation scheme, contextual-bag-of-words [22], to exploit named entity information. We further improve the performance by extracting likely positive and likely negative data from unlabeled data to provide additional training data. The performance of our GM and PPI text classification system is in the first quartile of the BioCreative II GM task (see the review by Smith and coworkers [12] included in this supplement). Our services support high-throughput online data processing and can be accessed online (see Table 1) and as an XML-RPC service at [23].

#### Division of Computational Bioscience, CIT, NIH (WL and CJ) [Lau]

GIANT (Gene Identification and Normalization Tool) is a rule-based system that uniquely identifies human gene mentions in free text [24]. The process is divided into two major steps. The goal of the first step is to extract all the potential gene mentions from the input text. Using a set of regular expression rules, gene symbols are detected using pattern matching. An approximate term searching technique is employed for gene names to account for typical morphological variations, such as word ordering. In the second step a set of statistical and heuristic features is used to estimate the level of confidence for each mention extracted. The confidence score is essentially a weighted linear combination of individual feature scores. The feature weights are optimized using the Nelder-Mead simplex method [25]. Precision of the result is improved by filtering out mentions with low confidence scores. The system has an F measure of 0.7622 from evaluations against the BioCreative II datasets. GIANT is implemented in Java and can be accessed either through a web interface or by remote procedure calls. The system stores a local copy of the Medline collection in a relational database.

#### Department of Computer Science, University of Tokyo (RS, KY and YC) [Sætre]

The AKANE++ system is a recently developed sentence-level PPI system. In order to use the AKANE system for the BioCreative tasks, the output format had to be simplified, because BioCreative just considers whether the abstract level contains interacting protein pairs or not. The original format of the AKANE system used annotated sentences like those in the AImed corpus [26]. In the new system, the abstracts are sent through a processing pipeline, containing modules for sentence splitting, tokenization and parsing, and then each mention of protein names are tagged by a named entity recognizer and normalized to their UniProt Identifiers. Finally, co-occurring pairs in single sentences are used as candidates for the PPI classification system. Some simple postprocessing is done in order to transform the sentence-level results from the AKANE system into the expected format for the BioCreative II challenge. The postprocessing included filtering and ranking of the sentence-level results, and then deciding whether the collective PPI confidence was high enough to assume that the abstract contains PPI interactions [27]. A separate system developed by our team is the ProtIR, filtering and ranking articles by their PPI relevance, based on a bag-of-words IR approach. Further details can be found in [28].

#### Biointelligence Laboratory, Seoul National University (SK, SS and BZ) [Kim]

Our PIE (Protein Interaction Extraction) system was developed to identify the PPI information from biomedical literature. The system consists of two modules for PPI article filtering and PPI sentence filtering. Each module uses ML techniques, and performs the filtering tasks based on the idea that the PPI descriptions have their own patterns at the article and sentence levels [29]. For the meta-services, the PPI article filter is utilized to support PPI classifications from PubMed abstracts and full text. The article filter uses a cost-sensitive learning algorithm, AdaCost [30], combined with the naïve Bayes classifiers. Unlike other ML-based classifiers minimizing the number of incorrect classifications, AdaCost provides the flexibility of controlling the precision and recall rates by means of a cost factor. In addition, naïve Bayes classifiers can easily take into account heuristic knowledge in a probabilistic form. For the AdaCost algorithm, a document is preprocessed by stemming and stopword removal. We use a modified stopword list (available at [31]), where the PPI-related words are omitted from common stopwords. Then, the remaining sentences are converted into the bag-of-words representation to discover the specific words or combinations of the words that best capture the PPI relevance at the article level.

#### Center for Computational Pharmacology, University of Colorado (WB and LH) [Baumgartner]

The Center for Computational Pharmacology's Annotation Server provides gene mention and gene normalization annotation, and protein interaction classification functionality on both full-text and PubMed abstracts. Annotation output is generated using an integrated approach to concept recognition. Gene mentions are detected using a stochastic tagging system built and trained for the inaugural BioCreative challenge [32]. Gene normalization is achieved by matching gene mention text to a lexicon of gene names constructed from human Entrez Gene records. Features of the normalization system include use of multiple gene taggers as input, simple conjunction resolution, a heuristic regularization procedure for processing gene names, exact matching of gene names to the lexicon, and a disambiguation step for gene names that match to multiple Entrez Gene records. Protein interactions are classified using our BioCreative II IPS (interaction pair subtask) system, which uses a concept recognition system developed by our group, OpenDMAP, and a series of manually generated patterns to classify PPIs in text [33]. Future development of the annotation server will involve streamlining the various systems to facilitate faster processing as well as incorporation of our BioCreative II ISS (interaction sentence subtask) system and extending our GN system's ability to normalize to more than just human genes.

#### School of Informatics, University of Edinburgh (BH, MM and XW) [Haddow]

In the system from the University of Edinburgh, the gene mentions are found using a CRF-based named entity tagger trained on the BioCreative training data. The tagger employes contextual, shallow grammatical, and morphological features tailored to the biomedical domain, as well as a gazetteer of protein names derived from RefSeq. For gene normalization, each of these gene mentions is mapped to a set of possible UniProt identifiers selected from the lexicon using a modified version of the Jaro-Winkler string similarity function [34]. To choose the most likely identifier from the set, a ML-based disambiguator (trained on BioCreative data) and a species tagger (trained on in-house data) are employed. Taxonomy annotations are also provided by the species tagger. The articles containing PPIs are selected using a SVM classifier trained on the BioCreative training set, and using conventional bag-of-words features, as well as features derived from the output of our PPI pipeline [35-39].

#### Medical Informatics Service, University and Hospitals of Geneva (PR, FE) [Ruch]

Our approach is based on the combination of basic pattern matching methods, the use of specialized heuristics and database resources, and a generic text categorization engine. The first step consists of extracting protein names from the abstracts together with a targeted list of the interaction verbs. The second step consists of deciding which proteins should be selected in order to build the appropriate interaction pairs. Because we have to provide a UniProt ID (accession number) for every protein, during a third step we identify the different species that appear in the documents. Indeed, protein names are usually associated with several species and therefore they are highly ambiguous regarding the sequences that they refer to. In the following step, all of the information is combined to obtain a unique UniProt ID. Finally, the interactions are ranked based on a combined model that takes into account the following features: protein names, interaction verbs, species, and word distances between these different entities. Species categories are identified using an automatic text categorization framework [40,41]. Because it is often difficult to find specific NEWT species in texts, a mapping table is manually maintained to associate MeSH-based species with their equivalent in NEWT. When no species are found, the system assumes that the protein is a human protein. The GPSDB resource [42] is used to help identify protein names in textual contents. Optionally, the system can also provide information concerning the interaction methods using the PSI-MI controlled vocabulary and the previously mentioned generic categorization system [41].

#### Department of Electrical Engineering and Computer Science, University of Michigan (AÖ, GE and DR) [Özgür]

We provide annotations for identifying interaction relevant articles. Our approach is based on extracting interacting protein pairs and evidence sentences from the articles by using dependency parsing and SVMs. After segmenting a given article into sentences and tagging the protein names with Genia Tagger [43], we build the dependency parse trees of the sentences by using Stanford Parser [44]. From the dependency parse trees of the sentences, we extract the shortest paths between each protein pair. We define a kernel function based on the edit distance based similarity among the extracted dependency paths. We use this kernel function with SVM to classify each sentence as being an evidence for the interaction of a protein pair or not. We annotate an article as an interaction relevant article if it contains an evidence sentence for the interaction of a protein pair. Detailed information about our annotation system can be found in [45,46].

#### Yale University School of Medicine and Yale University (MK and TL) [Luong]

We believe that gene name identification is a modular process that involves term recognition, classification, and mapping [47]. Here, we focus on gene name mapping, and use an existing program (ABNER [48]) for gene name recognition and classification (entity recognition). We use a combination of two methods to map recognized entities to their appropriate gene identifiers (Entrez GeneIDs): the trigram method and the network method. Both methods require preprocessing, using resources from Entrez Gene, to construct a set of method-specific matrices. We first address lexical variation by transforming gene names into their unique trigrams (groups of three alphanumeric characters) and perform trigram matching against the preprocessed gene dictionary. For ambiguous gene names we additionally perform a contextual analysis of the abstract that contains the recognized entity. We have formalized our method as a sequence of matrix manipulations, allowing for a fast and coherent implementation of the algorithm [49].

#### iHOP: information hyperlinked over proteins (RH, AV and CS) [Hoffmann]

The iHOP information resource [50,51] selectively retrieves information that is specific to genes and proteins and summarizes their interactions and functions. The system supports filtering and ranking of extracted sentences according to significance, impact factor, date of publication, and syntactical properties. Entity recognition and annotation processes (GN) in iHOP are based on a dictionary approach to screen for synonyms of genes and proteins, MeSH terms, and chemical compounds. Synonym dictionaries are regularly compiled and updated from various resources (for example, NCBI and UniProt) and extended programmatically to account for orthographic variations specific to the type of entity or organism. Draft annotations from entity-specific annotator processes are integrated into a final annotation, where individual finding sites are evaluated for uniqueness (relative to the entire synonym space), quality (based on properties of the synonym and the immediate context), and confidence (based on context information in the complete document and meta information). All annotations are mapped to corresponding external databases.

## Discussion

The BCMS platform unites and standardizes access to textual information extracted by various IE systems, presenting the annotations and classifications in a consistent structure. It aims to provide a public protocol to annotate biomedical text at the most basic level. At this stage, the platform provides an interface to explore and extract some of the annotation data created during the BioCreative II challenge [7], namely the four annotation types described in the Annotation systems section (above), for all of the official training and test set abstracts (a total of 22,804 Medline citations, minus 44 expired records at the time of this writing). A basic web interface and a web service API have been created. The communications layer (the XML-RPC transactions) is fully developed. The system can be synchronized with the complete PubMed/Medline database. It may be stated that the initial setup has been done, allowing us to advance to a fully featured version of the platform once the current state is accepted by the community.

Although such a distributed IE system seems fairly simple, numerous obstacles needed to be solved, such as the following.

• One of the most obvious problems is data consistency. The PubMed database is a dynamic resource in which citations are not only added but also changed and deleted (see the annual 'Medline/PubMed update charts' [52]; this affects several tens of thousands of records per year and is occurring on a daily basis.

• A less obvious difficulty is string encoding. As with biological sequences, when talking about positions and offsets in the sequences, using different encoding schemas would produce different and ultimately erroneous data. Therefore, continuous use of Unicode is enforced.

• Special attention had to be paid to the communications layer, specifically between the meta-server and the annotation servers. This component is virtually separated from the meta-server and multithreaded to ensure consistent and unimpeded usage.

At this stage, the system provides a limited compilation of the data generated during BioCreative II, offering integrated access to the systems produced by some of the groups participating in the second BioCreative challenge. The platform at its current state is confined to the approximately 22,800 abstracts used during BioCreative II. The intent is to open the system up (most likely stepwise, to avoid massive overload of the annotation queues) to the complete set of Medline records, and we are considering allowing annotation of user-provided full text after the prototype stage has been completed. The development of a platform that can operate freely on the complete set of Medline abstracts will be of great advantage to the biomedical community. Therefore, the next step is to go from the prototype state with a limited set of abstracts to the open system, where users can obtain classifications for any PubMed citation.

## Conclusion

This prototype is the first meta-service for biomedical information extraction. The platform is based on design principles of simplicity and expandability. Future initiatives to expand the system, such as adding annotation types or opening the system for user-provided texts, are likely to be possible with little effort. This implies that other research groups can join the platform, providing their own annotations, including the expansion of the system for new annotation types, for example, for protein-interaction detection methods. The three main units of the system (the various annotation systems, the annotated data, and the access methods) as well as their components (data, communications, and application layer) are independent of each other, so that one of the parts can be manipulated or completely exchanged without affecting the platform as a whole.

Furthermore - and similar to the development of the meta-servers in the field of protein structure prediction [53], in which a central server collects the results of several structure prediction algorithms and unifies these to create a jury prediction on the sequence, delivering the result to the client - we foresee that this platform and possibly others would evolve to compare the various annotation systems. This is because a single dataset is returned for the complete annotations from all systems on a given abstract by using the web service interface, with calibrated annotations and systematic consensus annotations. It will be interesting to use these consensus annotations as a baseline for future BioCreative challenges.

## Materials and methods

Research groups interested in contributing to the BCMS platform are requested to contact the corresponding author for further specifications and sample implementation of an AS in Java.

### Data layer

The most critical element is the textual data *per se *(the PubMed/Medline records, which are represented using UTF-8 encoding). Both the meta-server and the ASs are required to have access to the same Medline records (data persistency and consistency). ASs are required to keep their local copy of Medline up to date on at least a weekly basis. There are various means by which this can be achieved, for instance by maintaining a local copy of the Medline database (FTP download) or using the eUtils service oriented architecture protocol (SOAP) API [54] to retrieve the record for the current request. The latter has the advantage of being up to date with the very latest state (data consistency) from Medline, but it also makes the AS dependent on the availability of the NCBI service. This online solution is the default provided by the sample AS implementation.

### Communications layer

For solving inter-server communications, as well as client to meta-server communications, the three most common web protocols were considered: representational state transfer (REST), XML-RPC, and SOAP. XML-RPC was chosen for the following reasons.

• General: it is the oldest protocol available, which means that it is the most widespread (many web libraries include this protocol by default) and best known, and can therefore be assumed to be robust.

• Versus REST: it has the advantage that message length is not limited, as opposed to REST, which uses the URL to transmit parameters. The general World Wide Web Consortium recommendation for web development is not to use URLs of more than 2,000 characters. These URL specifications would limit free text annotations.

• Versus SOAP: XML-RPC is much less complex, and the specification paper is about one-sixth of the SOAP specification. All functionality required for the platform is provided by the XML-RPC protocol already. Therefore, XML-RPC guarantees rapid and simple implementation and provides a straightforward means of debugging and maintaining the system.

In the current setup, teams providing annotations can either create their own XML-RPC server implementation (following the platform guidelines) or they can simply use the default AS system, which is a standard Java implementation based on the Apache XML-RPC library (because Java is the most common programming language and many sites use the Apache framework), which can be requested from the corresponding authors.

### Application layer

The following tools, libraries, and applications have been used to develop the platform.

• PostgreSQL 8.1: database for both Medline and the annotation data [55].

• Django 0.96/stable: WDF, embedded in Apache 1.3 [56].

• jQuery 1.2.1: AJAX and interactive webpage elements [57].

• LingPipe 3.2.0: download of and synchronization with the Medline database [58] (on a daily basis).

• Python 2.5: in-house implementation of the XML-RPC communications layer using the standard library [59].

The versions correspond to the latest used versions as of the time of this writing and are subject to change.

## Abbreviations

AS, annotation server; BCMS, BioCreative MetaServer; CRF, conditional random field; GIANT, Gene Identification and Normalization Tool; GM, gene mention; GN, gene normalization; IE, information extraction; iHOP, Information Hyperlinked Over Proteins; IR, information retrieval; ML, machine learning; NCBI, National Center for Biotechnology Information; PPI, protein-protein interaction; REST, representational state transfer; RPC, remote procedure call; SOAP, service oriented architecture protocol; SVM, support vector machine.

## Competing interests

The work of BH, MM, and XW was funded by ITI Life Sciences, Scotland, whose mission is to explore commercialization of promising technologies in the life sciences. All other authors declare that they have no competing interests.

## Authors' contributions

AV and MK: BioCreative II workshop. AV, CRP and MK contributed to meta-server system design. FL: meta-server system design and implementation. AV: supervisor at CNIO. All others: annotation servers and systems. Author names were sorted by institution/university names except for CNIO authors. All authors have read and approved the final manuscript.
